# Alteration of resveratrol-dependent glycosyltransferase activity by elicitation in DJ-526 rice

**DOI:** 10.1080/21645698.2020.1859314

**Published:** 2021-01-04

**Authors:** Vipada Kantayos, Jin-Suk Kim, So-Hyeon Baek

**Affiliations:** Department of Well-being Resources, Sunchon National University, Suncheon, Korea

**Keywords:** Glycosyltransferase, resveratrol, GM rice, seed germination, gene expression

## Abstract

Since the successful creation of DJ-526, a resveratrol-enriched rice cultivar, research has focused on resveratrol production because of its great potential in pharmaceutical applications. However, the utilization of resveratrol in DJ-526 is limited by glycosylation, which converts resveratrol to its glucoside (piceid), in a process driven by glycosyltransferase. The verification of resveratrol-dependent glycosyltransferase activity is an essential strategy for improving resveratrol production in DJ-526 rice. In this study, 27 candidate glycosyltransferases were evaluated in germinated seeds. Among the candidates, only R12 exhibited upregulation related to increased resveratrol and piceid content during seed germination, whereas various effects on the activity of glycosyltransferase were observed by the presence of a bio-elicitor. Yeast extract tended to enhance glycosyltransferase activity by seven candidates, and a specific peak for an unknown compound production was identified. Conversely, chitosan acted as a glycosyltransferase inhibitor. Our results suggested that R12 and R19 are the most relevant candidate resveratrol-dependent glycosyltransferases in DJ-526 seeds during germination and elicitation. Future research should assess the possibility of silencing these candidate genes in an effort to improve resveratrol levels in DJ-526 rice.

## Introduction

Resveratrol (3,5,4-trihydroxy-trans-stilbene) has been widely studied for its biological and pharmaceutical activities, as the compound exhibits various effects including anti-oxidant, anti-cancer, anti-fungal, and anti-aging activity. Resveratrol is generally found in several plants including grapes, peanuts, and berry fruits, and the demand for resveratrol-containing products in the natural product market is increasing. Genetic modification has been used to develop plant-derived natural products, which could create new commercial opportunities for resveratrol-derived crops. In a previous study, DJ-526 rice was successfully developed from the original ‘Dongjin’ cultivar by inserting the gene encoding stilbene synthase, a key enzyme for resveratrol synthesis, in an effort to produce resveratrol in rice seedlings. Both the callus and seeds of DJ-526 have been reported to have biological and pharmaceutical activities, e.g., cholesterol reduction,^1^ whitening effects,^2^ extension of the life cycle of fruit flies,^3^ improvement of physical strength, and anti-aging properties.^4^

However, the utilization of resveratrol is limited by glycosylation, which is a congenital process in plants. Synthesized resveratrol is naturally transformed to its glucoside form and stored in vacuoles to prevent self-toxicity and oxidation.^5^ The conversion of resveratrol to its glycoside (piceid) is generally catalyzed by glycosyltransferase, which transfers a glycosyl group from the sugar donor to the specific acceptor (aglycone, resveratrol), thereby forming the modified structure (piceid).

In rice, glycosylation is an important reaction that allows sugar moieties to attach to molecules such as proteins, lipids, nucleotides, and other small molecules to modulate charges and increase chemical stability in cells.^6^ The most well-known sugar donor is UDP-glucose, which participates in carbohydrate metabolism during rice seed development.

The use of elicitors was reported to improve the yield of resveratrol. Biotic elicitors such as polysaccharides including chitosan and alginate have been used to induce resveratrol production in cell cultures of *Vitis vinifera*.^7^ Similarly,,^8^indicated that chitosan can enhance the expression of proteins related to stilbene synthesis. Yeast extract has been used to stimulate the production of secondary metabolites such as phenolic compounds^9^ by more than 1.5-fold in the case of ginsenoside.^10^ However, most previous research studied cell suspensions of *Vitis vinifera*, which is not a genetically modified (GM) source.

This study is the first report on the effect of yeast extract and chitosan as elicitors on the production of resveratrol and piceid in an effort to identify candidate resveratrol-dependent glycosyltransferases in the resveratrol-producing GM rice cultivar DJ-526. The relationship between the conversion of resveratrol to piceid was analyzed using HPLC, and the expression of resveratrol-related glycosyltransferases was examined via real-time PCR.

## Materials and Methods

### Plant Materials and Germination Medium Preparation

DJ-526 and Dongjin (non-GM) rice cultivars were provided by the Rural Development Administration of Korea. Seeds were sterilized with 70% (v/v) ethanol and 5% (v/v) of sodium hypochlorite for 1 h. Autoclavable plant growth regulators-free agar medium was used as germination medium for prevention of synthetic compound interference. Seeds were harvested on 1, 3, 5, and 7 days after culture on agar media for High-performance liquid chromatography analysis and gene expression analysis.

### Elicitor Preparation

Both yeast extract and chitosan were examined as elicitors in 5-day-old seedlings because of the increased glycosylation in germinated seeds (without treatment). The seeds from the germination date (5 days) were cultured under various concentrations and elicitation times. Bacto™ yeast extract from autolyzed yeast cells (Becton, Dickinson and Company) were prepared at concentrations of 1, 3, and 5 g/L via dilution with distilled water and autoclaved before use. Likewise, chitosan solutions were prepared at the same concentrations used for the yeast extract by dissolving medium molecular weight chitosan (Sigma-Aldrich) in 1% acetic acid. Seedlings were cultured with elicitors for 6, 12, or 24 h.

### Quantification of Resveratrol and Piceid Content

Harvested samples were dried in an oven at 60°C for 24 h or until the dry weight was stable. The dried sample (0.1 g) was macerated with 1 mL of 80% methanol and then placed in an ultrasonic bath (Jinwoo-Alex Company, Korea) at 40°C for 30 min. The fluid extract was filtered through a 0.45-μm nylon filter (Hyundai Micro Co., Ltd.). The resveratrol and piceid content during germination and in the presence of elicitors was determined using an HPLC system (Waters separation module e2695) with a reverse-phase column (C18, 4.6 × 150 mm, Waters). The mobile phase consisted of 100% acetonitrile as solvent A and water as solvent B, and the solvents were transported at a flow rate of 1 mL/min.

A gradient elution program was carried out by changing the ratio of solvent A and solvent B, from 10: 90, solvent A: B for 37 min, then 30: 70 for 1 min, 100: 0 for 7 min. then return to the initial condition 10:90 for 5 min. The UV-visible detector was operated at 308 nm. Trans-piceid and trans-resveratrol standards were obtained from Sigma-Aldrich. We analyzed piceid and resveratrol levels simultaneously using a standard mixture of piceid and resveratrol. The peaks of piceid and resveratrol were observed at retention times of approximately 16.2–16.8 and 26.7–27.3 min, respectively. The P:R ratio was determined to represent glycosyltransferase activity. In this ratio, P represents the piceid content, and R denotes the resveratrol content. If the P:R ratio exceeds 1, then it is assumed that glycosyltransferase can actively convert resveratrol to piceid, whereas a P:R ratio of less than 1 indicates that glycosyltransferase activity is suppressed because the resveratrol concentration is lower than the piceid concentration.

### RNA Extraction and cDNA Synthesis

RNA of germinated DJ-526 and Dongjin seeds (non-transgenic seeds) was extracted using an IQeasy™ plus plant RNA extraction kit (Intron). The quantity of RNA was assessed using a Spectramax microplate reader, and gel electrophoresis was used to confirm RNA integrity. cDNA was synthesized using ReverTra Ace® qPCR RT Master mix with gDNA removal (TOYOBO).

### Quantitative Real-time PCR

Real-time PCR was performed using a CFX Connect Real-Time PCR Detection System (Bio-Rad). The reaction was performed using RealMOD™ Green SF 2X qPCR mix (Intron) with a total reaction volume of 20 μL. Primers for candidate resveratrol-dependent glycosyltransferase genes were designed by referencing nucleotide information from the NCBI database (Table 1). The concentration of each candidate primer was 5 pmol, and *β-actin* was used as an internal reference gene. Based on the HPLC data, germinated 1- and 5-day-old seedlings were used to analyze glycosyltransferase expression. In addition, 5-day-old seedlings were treated with 3 g/L yeast extract or chitosan for 6 or 24 h to examine gene expression.

**Table 1.** Twenty-seven candidate resveratrol-dependent glycosyltransferases

No.	Gene symbol	Gene ID	Description
1	R1	LOC4340967	Anthocyanidin 5,3-*O*-glucosyltransferase
2	R2	LOC4330773	7-deoxyloganetin glucosyltransferase (leaves, roots)
3	R3	LOC4331831	UDP-glycosyltransferase TURAN (all organs)
4	R4	LOC4341323	UDP-flavonoid 7-*O*-glucosyltransferase 73C6 (all organs) UDP-glycosyltransferase TURAN
5	R5	LOC4328853	UDP-glycosyltransferase 72B1 (flowers, leaves)
6	R6	LOC4328677	UDP-glycosyltransferase 73C3 (leaves, roots, seeds)
7	R7	LOC4326220	UDP-flavonoid 7-O-glucosyltransferase 73C6 (all organs)
8	R8	LOC4347591	UDP-glycosyltransferase 74F2 (leaves, roots, seeds) indole-3-acetate beta-glucosyltransferase
9	R9	LOC4335169	UDP-glycosyltransferase 74F2 (all organs)
10	R10	LOC4335168	UDP-glycosyltransferase 74F2 (seeds)
11	R11	LOC4335166	UDP-glycosyltransferase 74E2 (all organs) indole-3-acetate beta-glucosyltransferase
12	R12	LOC4336447	UDP-glycosyltransferase 82A1 (roots, flowers, all organs)
13	R13	LOC4334167	UDP-glycosyltransferase 83A1 (all organs)
14	R14	LOC4348311	UDP-glycosyltransferase 83A1 (roots, leaves, seeds, all organs)
15	R15	LOC4339543	UDP-glycosyltransferase 84A3
16	R16	LOC4345521	UDP-glycosyltransferase 85A2 (flowers, leaves, roots)
17	R17	LOC4348729	UDP-glycosyltransferase 86A1 (roots, seeds, leaves) UDP-glycosyltransferase 86A2
18	R18	LOC4327545	UDP-glycosyltransferase 87A1(seeds, leaves, roots) UDP-glucose:salicylic acid glucosyltransferase
19	R19	LOC4327546	UDP-glycosyltransferase 87A2 (seeds, all organs)
20	R20	LOC4339389	UDP-glycosyltransferase 88B1 (flower buds) UDP-glycosyltransferase 88F3
21	R21	LOC4339394	UDP-glycosyltransferase 88F5 (leaves, flowers)
22	R22	LOC4337226	UDP-glycosyltransferase 89B1 (roots, flower, all)
23	R23	LOC4345896	UDP-glycosyltransferase 89B1 (roots, leaves, all)
24	R24	LOC4335428	UDP-glycosyltransferase 90A1
25	R25	LOC4333838	UDP-glycosyltransferase 91B1
26	R26	LOC4335348	UDP-glycosyltransferase 92A1 (leaves, flower, roots, all organs)
27	R27	LOC9266253	UDP-glycosyltransferase 708A6; UDP-glycosyltransferase-like protein

### Statistical Analysis

Each experiment was repeat in triplicate. The resveratrol and piceid content of DJ-526 under elicitor treatment and without treatment were expressed as mean ± standard deviation (SD) by *P-values* less than 0.05 (*p* < .05) indicated statistically significant. The correlation between resveratrol and piceid level was analyzed by Pearson’s correlation coefficient using Excel 2016 (Microsoft).

## Results

### The Alteration of Resveratrol and Piceid Content in Germinated DJ-526 Seeds

**Figure 1.**
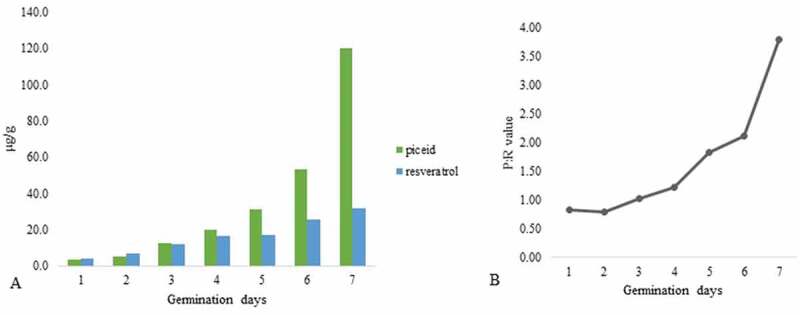
Changes of resveratrol and piceid content during the germination of DJ526 seeds for 1–7 days. The methanol extract of each sample was analyzed for resveratrol and piceid content via HPLC. (a) Graph presenting the germination period (x-axis) and changes of the piceid and resveratrol concentrations (y-axis). (b) Resveratrol-dependent glycosyltransferase activity was represented as the P:R ratio. P:R > 1 indicated that the glycosylation of resveratrol to piceid (resveratrol-dependent glycosyltransferase) was activated, whereas P:R < 1 indicated that glycosylation was suppressed

Piceid and resveratrol content significantly increased with increasing germination time (Fig. 1). Before germination (day 0), the piceid and resveratrol levels in DJ-526 rice seeds were approximately 3.5 and 1.4 μg/g, respectively. The yields of piceid and resveratrol in seedlings after 1–7 days of germination ranged 3.20–120.03 and 3.85–31.65 μg/g, respectively.

**Figure 2.**
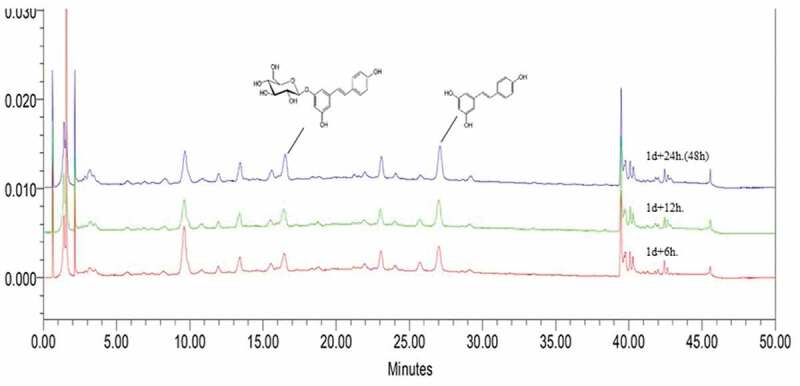
Chromatograms of piceid and resveratrol content during the second day of germination. Piceid and resveratrol were detected at retention times of 16 and 27 min, respectively. Between 6 and 12 h on day 1 of germination, piceid and resveratrol content showed non-differentiable yield. The levels of both compounds were obviously increased after 48 h (2 full days of germination). The red arrow denotes an unknown peak that may be related to the growth and development of rice seeds. The size of the peak decreased over time

The resveratrol and piceid content did not differ significantly between 6 and 12 h on the first day of germination (Fig. 2), but the resveratrol level on day 2 of post-germination

was noticeably higher than the piceid level, resulting in a P:R ratio of 0.78. However, on day 3 of germination, the piceid content was increased, and the piceid concentration was 2- and 4-fold higher than the resveratrol concentration on days 5 and 7 of germination, respectively.

The piceid and resveratrol content during 7 days of germination was significantly and positively correlated (*r* = 0.92). Meanwhile, we noticed an unknown peak at approximately 9.8 min related to the growth and development in DJ-526 seeds. This peak was evident on the first day, and it gradually diminished over time (Fig. 2)

### Effect of Yeast Extract and Chitosan on Resveratrol and Piceid Levels

**Figure 3.**
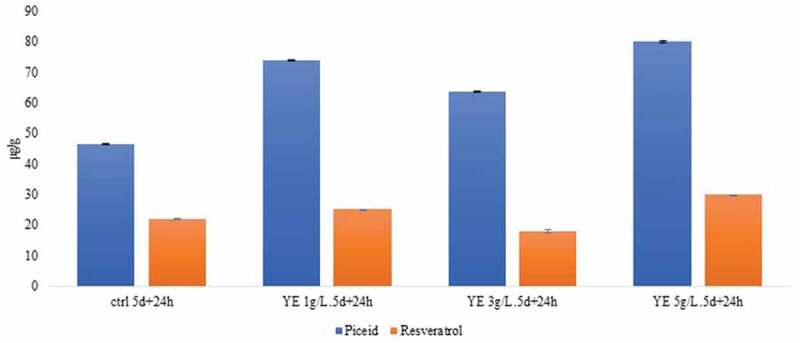
Bar graph presenting the piceid and resveratrol levels in 5-day-old seedlings cultured in yeast extract for 24 h. Each bar represents the mean±SD in triplicate

Five-day-old seedlings were cultured with different concentrations of yeast extract and chitosan for various intervals. The concentration of piceid in untreated 5-day-old seedlings ranged 41.9–55.5 μg/g, and the resveratrol content ranged 19.4–25.5 μg/g (Fig. 3) Yeast extract exposure for 24 h resulted in a substantial effect on piceid content compared with the findings in the untreated control, whereas no such increase was noted after 6 or 12 h of elicitation. After 24 h of yeast extract elicitation, the piceid concentration ranged 63.6–80.0 μg/g. Conversely, the resveratrol concentration after this treatment ranged 17.9–29.9 μg/g, which was not significantly different from the control levels. We found that yeast extract at a concentration of 3 g/L enhanced piceid levels while exerting exerted no effect on resveratrol levels, and the correlation between resveratrol and piceid was significant (*r* = −0.86, *p* < .05). This concentration was selected to verify the expression of resveratrol-dependent glycosyltransferase genes.

**Figure 4.**
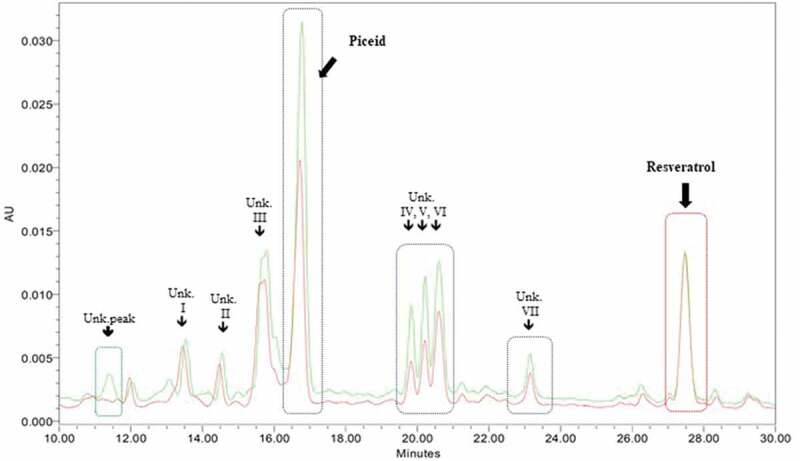
HPLC chromatogram of the methanol extract of 5-day-old germinated seeds elicited with 3 g/L yeast extract for 24 hours versus the untreated control. The retention time of piceid was 16.2 min, and resveratrol was detected at 26.6 min. After 5 days, piceid content was increased compared with the control level, whereas the resveratrol yield was indistinguishable from the control level

Although the chromatogram for 5-day-old seedlings displayed the same pattern in the control and 3 g/L yeast extract groups, a peak appeared at 11.4 min in the 3 and 5 g/L yeast extract treatment groups that was not present in the control or 1 g/L yeast extract chromatograms (Fig. 4). Moreover, we specifically detected several altered peaks, including unknown peaks IV, V, and VI, which were specific for seed germination under the light condition (Fig. 4) compared with the dark condition (data not shown).

**Figure 5.**
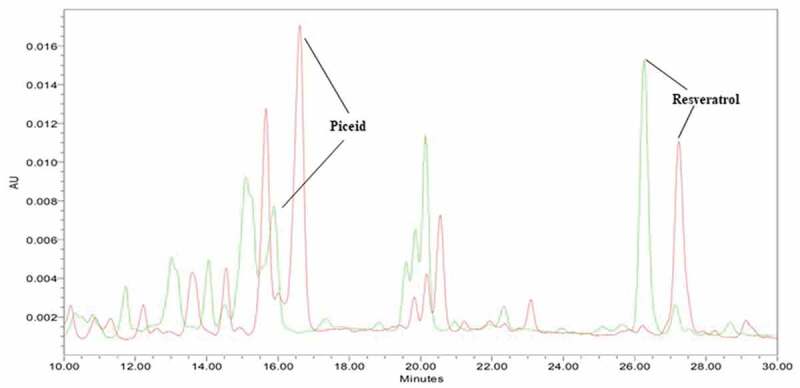
Chromatogram presenting piceid, resveratrol, and unknown peaks in 5-day-old germinated DJ526 seeds. DJ526 seedlings were treated with 1 g/L chitosan for 6 (red line) or 24 h (green line)

In case of chitosan, which acts as glycosyltransferase inhibitor, decreased the levels of piceid at all concentrations, increasing of elicitation time results in piceid reduction while resveratrol content (Fig. 5).

### Selection of Candidate Resveratrol-dependent Glycosyltransferases in Germinated DJ526 Seeds

The expression of candidate glycosyltransferases was evaluated during germination and in the presence of elicitors. Based on the HPLC results in which the P:R ratio was twofold higher on day 5 of germination than on day 1, seeds germinated for 1 and 5 days were selected to assess gene expression. Appropriate primers were determined using Cq values, and a single sharp melting curve (indicating specific amplification) was identified among the 27 candidate genes. Among the candidate glycosyltransferases, only R12 was upregulated in germinated DJ-526 seeds on day 5, suggesting its function as a candidate resveratrol-dependent glycosyltransferase gene.

**Figure 6.**
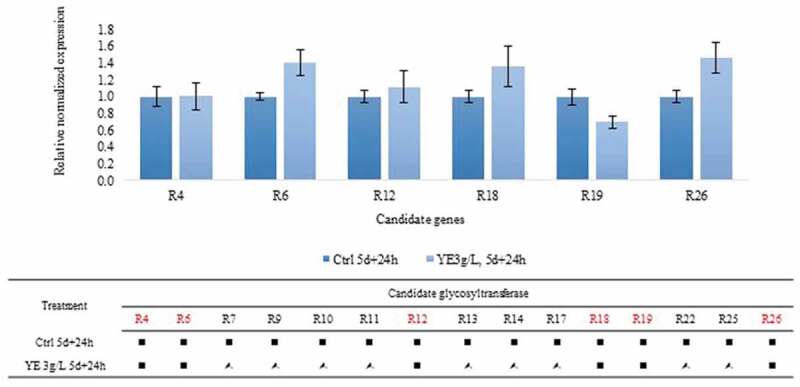
Relative expression of candidate glycosyltransferase genes presented as the mean ± SEM. The normalized fold change was evaluated following elicitation with 3 g/L yeast extract for 24 h versus the control. (■) Candidate genes with no change in expression. (*) Upregulated candidate genes. According to the HPLC data (Fig. 4), no change was found in resveratrol content therefore candidate genes that display consistent expression (no change in expression) would be the resveratrol-dependent glucosyltransferase. Each determination was calculated as the relative expression by Bio-rad CFX Maestro software

We also measured glycosyltransferase expression in the non-GM rice cultivar Dongjin and found the same expression pattern in DJ-526, namely upregulation of only R12 (data not shown). Concerning elicitor-treated germinated seeds, yeast extract was used at a concentration of 3 g/L, and 5-day-old seedlings were applied to study the expression of glycosyltransferases. According to the HPLC data, the piceid content increased after a long period of elicitation, whereas the resveratrol content remained unchanged. The results illustrated that the expression of seven candidate glycosyltransferases, namely R3, R4, R6, R12, R18, R19, and R29, was not changed compared with the control levels based on a fold change threshold of 1.5 (Fig. 6), whereas nine candidate glycosyltransferases, including R7, R9, R10, R11, R13, R14, R17, R22, and R25, were upregulated.

**Figure 7.**
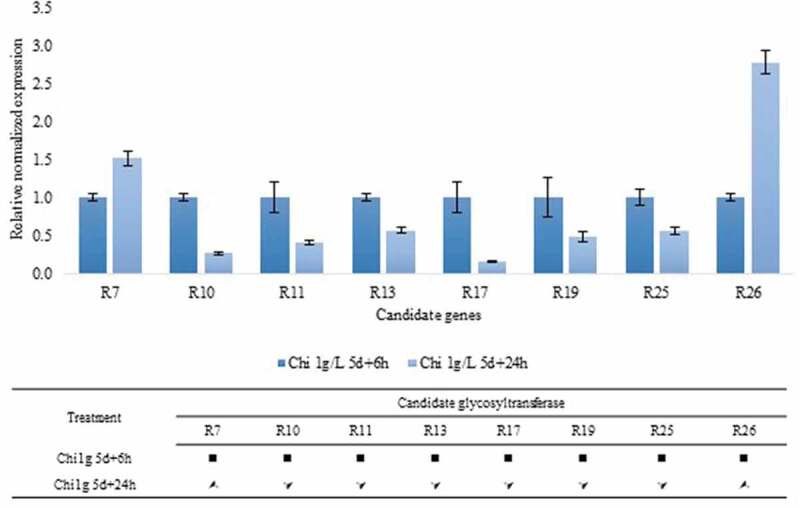
Bar graph presenting the downregulation of candidate glycosyltransferases following elicitation with 1 g/L chitosan for 6 or 24 h. Gene expression was assessed in 5-day-old seedlings. (■) Candidate genes with no change in expression. (*) Upregulated candidate genes. (*) Downregulated candidate genes

In the presence of 1 g/L chitosan, piceid levels were decreased according to the elicitation time; conversely, the concentration of resveratrol was increased, indicating that the glycosyltransferase responsible for piceid production was downregulated. Downregulation was observed for six candidate genes, namely R10, R11, R13, R17, R19, and R25 (Fig. 7), whereas R7 and R26 were upregulated. Other candidate genes displayed no change in expression versus the control. Concerning the alteration of piceid–resveratrol production in relation to glycosyltransferase expression, R12 and R19 exhibited the same expression patterns in both germinated seeds and elicitor-treated seeds. Thus, these genes were identified as candidate resveratrol-dependent glycosyltransferases.

## Discussion

The germination of rice seeds was initiated by placing the seeds on sterilized agar media or after an inhibition process. We used only 7 g/L agar and distilled water as the germination media without other chemical reagents to avoid interference caused by other compounds. Generally, seeds accumulate water in the first 20 h without morphological changes, and then metabolic and biochemical changes are consecutively induced by the increasing respiration rate.^11^ Accordingly, not all seeds are germinated, and we only harvested germinated seeds for the experiment. However, the germination efficiency depends on various factors, e.g., germination conditions and genotype.^12^

Our study revealed that the piceid and resveratrol content in germinated DJ-526 seeds increased with increasing germination time. The concentration of resveratrol in DJ-526 before germination was similar to that reported for DJ-526 rice (approximately 1.4–1.9 μg/g).^13^ Bioactive compounds are accumulated at different levels. For example, in two different rice cultivars, developing seeds displayed higher total phenolic and flavonoid levels than mature seeds.^14^ Hence, optimal growth and development conditions would result in the production of favorable bioactive compound amounts in the plant.

In the present study, piceid was present in rice at substantially higher levels than resveratrol after 7 days of germination. Similarly,,^15^revealed that piceid is generally present at 2–4-fold higher levels in wine than resveratrol^16^ and more than twofold higher levels in *Polygonum cuspidatum*.^17^

The conversion of resveratrol to piceid glucoside is driven by glycosyltransferase.^18^ During growth and development, the structure of the plant molecule is modified to maintain the cell balance and increase cell transport capacity.^19^ In this study, resveratrol levels were improved in DJ-526 seeds using elicitors including yeast extract and chitosan.

Yeast extract was demonstrated to elicit piceid production, and a negative correlation was observed between piceid and resveratrol content. One possibility is that piceid can be produced from other stilbenes. In addition, based on the unknown peak observed at 11.4 min following yeast extract treatment at higher concentrations, a specific compound or piceid–resveratrol derivative produced via glycosylation from yeast extract may have been present. It can be concluded that the yeast extract concentration may influence its specific effects on piceid production.

Meanwhile, chitosan has been used as an activator of defense mechanisms to improve metabolite levels in plants.^19–21^ However, chitosan exerted weaker effects on stilbene and resveratrol production in some cases^22,23,24^ and inhibited growth and development, which may be related to the production efficiency of bioactive compounds.^23^ Thus, the optimal concentration, elicitor, and duration of exposure to elicitors are important factors for bioactive compound production through growth and development or directly through their biosynthetic pathways.

The expression of resveratrol-dependent glycosyltransferases was investigated using HPLC data. The same expression pattern was observed for the candidate glycosyltransferase genes in 1- and 5-day-old germinated seeds of DJ-526 and Dongjin rice. From this result, we determined that these candidate genes may not be specific for resveratrol-dependent glycosyltransferases, and a gene that can regulate the production of various compounds at the transcriptional or metabolic level might exist.

According to our studies, increases in resveratrol and piceid content are attributable to the upregulation of a glycosyltransferase, as piceid is a major derivative of resveratrol. DJ-526 germinated seed in 5-day-old seedling showed up-regulation only in R12 related to yeast extract treatment which R12 exhibited no-change of glycosyltransferase expression following the HPLC data which found unchangeable of resveratrol content and the rest up-regulated candidates including nine candidates would relate to piceid-end product glycosylation by other stilbenes relative compound. Notwithstanding, under treatment with yeast extract or chitosan, R12 and R19 appear to be most relevant as candidate resveratrol-dependent glycosyltransferases.

## Conclusion

Resveratrol and piceid levels were sequentially increased during rice seed germination, and piceid levels were higher than resveratrol levels. However, the increase of piceid content did not always correspond to changes of resveratrol levels because piceid can be produced from other stilbenes. Furthermore, yeast extract elicitation increased piceid and resveratrol production in DJ-526 seeds, whereas chitosan inhibited resveratrol and piceid production. Among 27 candidate glycosyltransferases, R12 and R19 were identified as potential resveratrol-dependent glycosyltransferases based on their significant correlations with changes in resveratrol and piceid levels. The identification of candidate glycosyltransferases is important for improving resveratrol production in DJ-526 rice, which could have pharmaceutical benefits, although further study is required.
